# Discriminating Sleep From Freezing With Cortical Spindle Oscillations

**DOI:** 10.3389/fncir.2022.783768

**Published:** 2022-03-24

**Authors:** Marco N. Pompili, Ralitsa Todorova

**Affiliations:** ^1^Aix Marseille University, INSERM, Institut de Neurosciences des Systèmes (INS), Marseille, France; ^2^Department of Neurobiology and Behavior, Cornell University, Ithaca, NY, United States

**Keywords:** sleep spindles, behavioral state classification, freezing, sleep, cortical oscillations, local field potential oscillations

## Abstract

*In-vivo* longitudinal recordings require reliable means to automatically discriminate between distinct behavioral states, in particular between awake and sleep epochs. The typical approach is to use some measure of motor activity together with extracellular electrophysiological signals, namely the relative contribution of theta and delta frequency bands to the Local Field Potential (LFP). However, these bands can partially overlap with oscillations characterizing other behaviors such as the 4 Hz accompanying rodent freezing. Here, we first demonstrate how standard methods fail to discriminate between sleep and freezing in protocols where both behaviors are observed. Then, as an alternative, we propose to use the smoothed cortical spindle power to detect sleep epochs. Finally, we show the effectiveness of this method in discriminating between sleep and freezing in our recordings.

## 1. Introduction

One of the main ambitions of systems neuroscience is to understand the relationship between the activity of the nervous system and animal behavior. To this end, a fruitful approach is to record neural activity as animals perform specific tasks as well as during natural behavior. Indeed, monitoring brain activity across a wide range of training-dependent and spontaneous behavioral states is crucial in the study of potential mappings between neural activity and behavior (Krakauer et al., 2017). Moreover, contrasting the neurophysiology of active vs. inactive states (such as rest), helps disentangle the neural activity underpinning cognitive functions such as learning from those controlling motor behavior and perception. In particular, the neurophysiology of sleep examines endogenous activity taking place when the brain is disengaged from the external world and self-organized computation emerges (Buzsáki et al., 2014).

In electrophysiological studies, neural activity can be recorded for many hours, and in some cases days or weeks, across many behavioral states (e.g., Wilson and McNaughton, 1994; Hirase et al., 2001; Lin et al., 2006; Benchenane et al., 2010; Hengen et al., 2016; Dhawale et al., 2017; Girardeau et al., 2017; Chung et al., 2019; Todorova and Zugaro, 2019). Matching specific neural processes to particular behaviors requires reliable automated means to distinguish between different behavioral states, such as wakefulness vs. sleep, but also between rapid eye movement (REM) sleep and non-REM sleep, also known as slow-wave sleep (SWS).

State of the art automated brain state scoring techniques rely on motor activity as well as neural recordings, taking advantage of the fact that active awake behavior and REM are characterized by oscillations in the theta frequency band (7–8.5 Hz), while SWS is dominated by slower rhythms in the delta range (1–4 Hz). The most common approach for classifying sleep stages consists of sampling animal movement (using video recordings, inertial sensors, or electromyography) to detect immobility periods, and then dividing the resulting epochs into REM and SWS based on the relative power of theta vs. delta oscillations in the electroencephalography or local field potential (LFP) recordings. This technique has been used for more than 40 years (Gottesmann et al., 1971; Kohn et al., 1974; Johns et al., 1977) and is still very popular today (e.g., Todorova and Zugaro, 2019; Sosa et al., 2020; Wang et al., 2020; Tingley et al., 2021).

A limitation of this approach is the assumption that all immobility corresponds to rest, uniquely composed of its sub-states: quiet wakefulness, SWS, and REM. However, certain behavioral paradigms may involve immobility which should not be equated to resting. In particular, immobility is also the main feature of freezing behavior (Blanchard and Blanchard, 1969), a widely used index of fear typically employed to probe for learning in fear conditioning (Fanselow and Poulos, 2005), a very popular paradigm in rodent behavioral research. This presents a problem for studies addressing the role of sleep in emotional regulation and fear memory consolidation, a research focus likely to become more and more popular since sleep neurophysiology is finally crossing paths with the investigation of emotional learning (Trouche et al., 2020).

In order to correctly discriminate between sleep and freezing in a behavioral protocol involving both behaviors, we developed a method based on spindle oscillations in cortical recordings. Spindle oscillations are characteristic of SWS (Steriade et al., 1993; Fernandez and Lüthi, 2020), which constitutes 90% or rodents sleep (Brankack et al., 2010). Here, after illustrating how standard approaches, by construction, cannot discriminate between freezing and sleep/rest states, we show how these can be disentangled by the smoothed spindle power. Finally, we validate that this approach was effective in detecting sleep and freezing epochs interspersed in a fear conditioning and extinction paradigm. The algorithm we propose requires solely the LFP from a single neocortical channel and a readout of the animal motor activity and is therefore a simple and effective solution to determine behavioral states in freely moving animal recordings.

## 2. Results

### 2.1. Standard Approach Fails to Distinguish Sleep From Freezing

**Figure 1:**
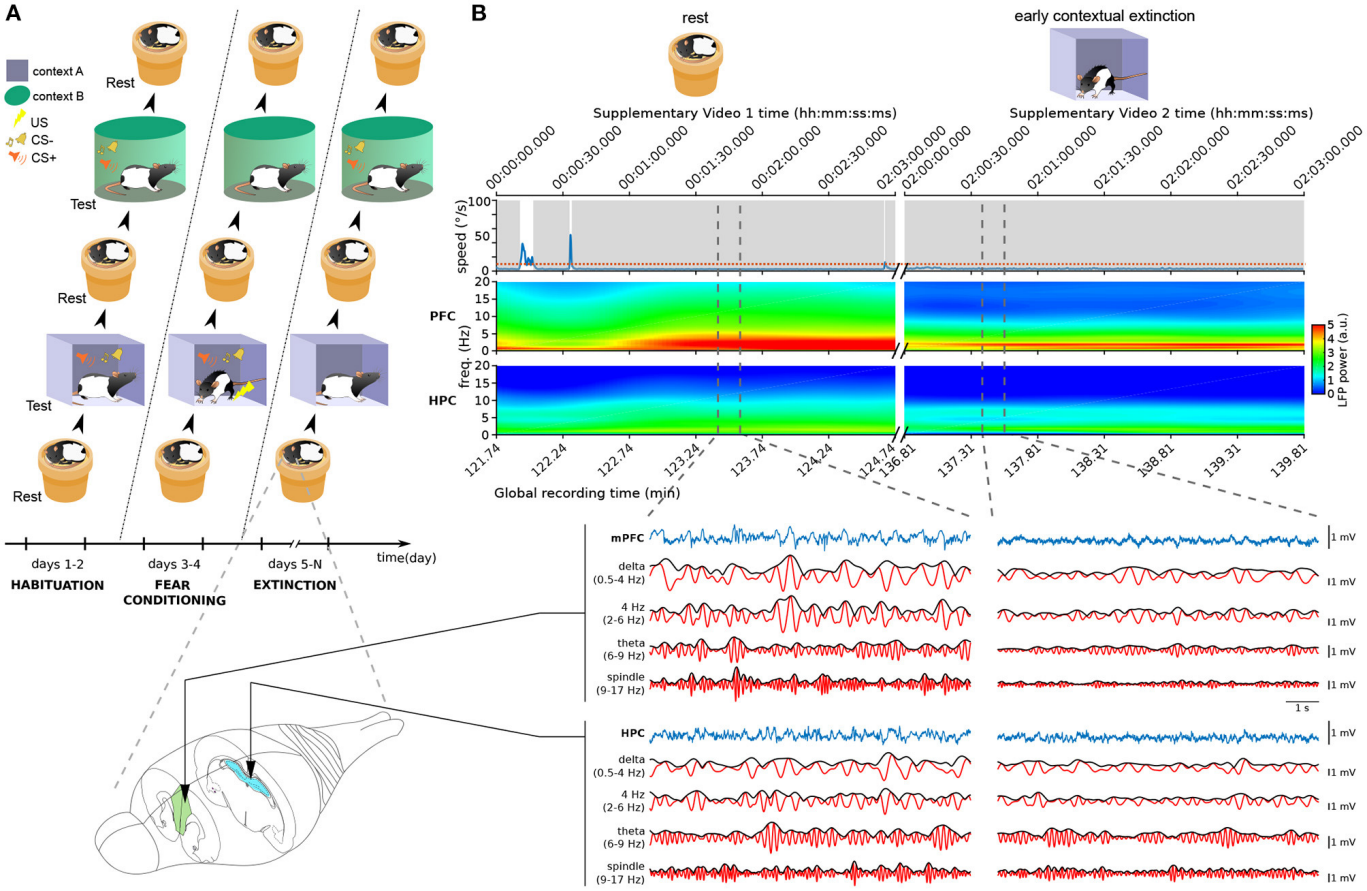
Neural and behavioral activity during rest vs. freezing **(A)** Overview of the behavioral protocol. Each day, animals were exposed to a resting environment (a familiar non-anxiogenic flowerpot) and to two testing environments **(A,B)**. In habituation sessions (2 days, left), animals were exposed to context A and context B and to the auditory stimuli employed in the protocol. This was followed by fear conditioning (2 days, middle) where the presentations of the conditioned stimulus (CS+) in context A were coupled with the delivery of aversive footshocks. In the following days of the protocol (6–12 days, right), the animals underwent contextual fear extinction in context A as well as cued fear extinction in context B (see Section 4). **(B)** Example neural activity recorded during sleep in the resting environment (left, see Supplementary Video 1) and during freezing in context A (right, Supplementary Video 2) in the first day of extinction training. Time stamps corresponding to those overlaid in Supplementary Video frames are shown on top. (Top) Animal speed as measured by head movements; red dotted line: threshold used to detect immobility; gray shadings: immobility periods. (Middle) Spectrograms of the LFP recorded in the medial prefrontal cortex (mPFC) and hippocampus (HPC). Vertical dashed lines: The boundaries of example intervals represented on the bottom. (Bottom) mPFC and HPC LFP signal recorded in the intervals marked above. Blue: raw recorded LFP; red: filtered LFP in the delta, 4 Hz, theta, and spindle frequency bands; black: instantaneous amplitude of the filtered LFPs.

As a case study, we analyzed data recorded from rats performing both freezing and sleeping behaviors. The rats underwent a multi-day fear conditioning and extinction protocol, and rest sessions were recorded in a familiar non-anxiogenic environment before and after each training session (Figure 1A).

We first used standard methods to detect freezing and sleep. Freezing is typically defined as all immobility periods longer than a particular threshold (0.5–2 s; e.g., Bagur et al., 2018; Moberly et al., 2018; Gründemann et al., 2019; Jercog et al., 2021; Ressler et al., 2021). Nonetheless, standard sleep-scoring algorithms assume that all such immobility corresponds to rest states. Therefore, these techniques label most immobility periods as both sleep and freezing. Indeed, while in some cases immobility periods correspond to sleep (see the left part of Figure 1B; Supplementary Video 1), a freezing period may also last for long periods of time (right part of Figure 1B; Supplementary Video 2). This makes the standard approach to sleep detection unsuitable for data involving freezing.

**Figure 2:**
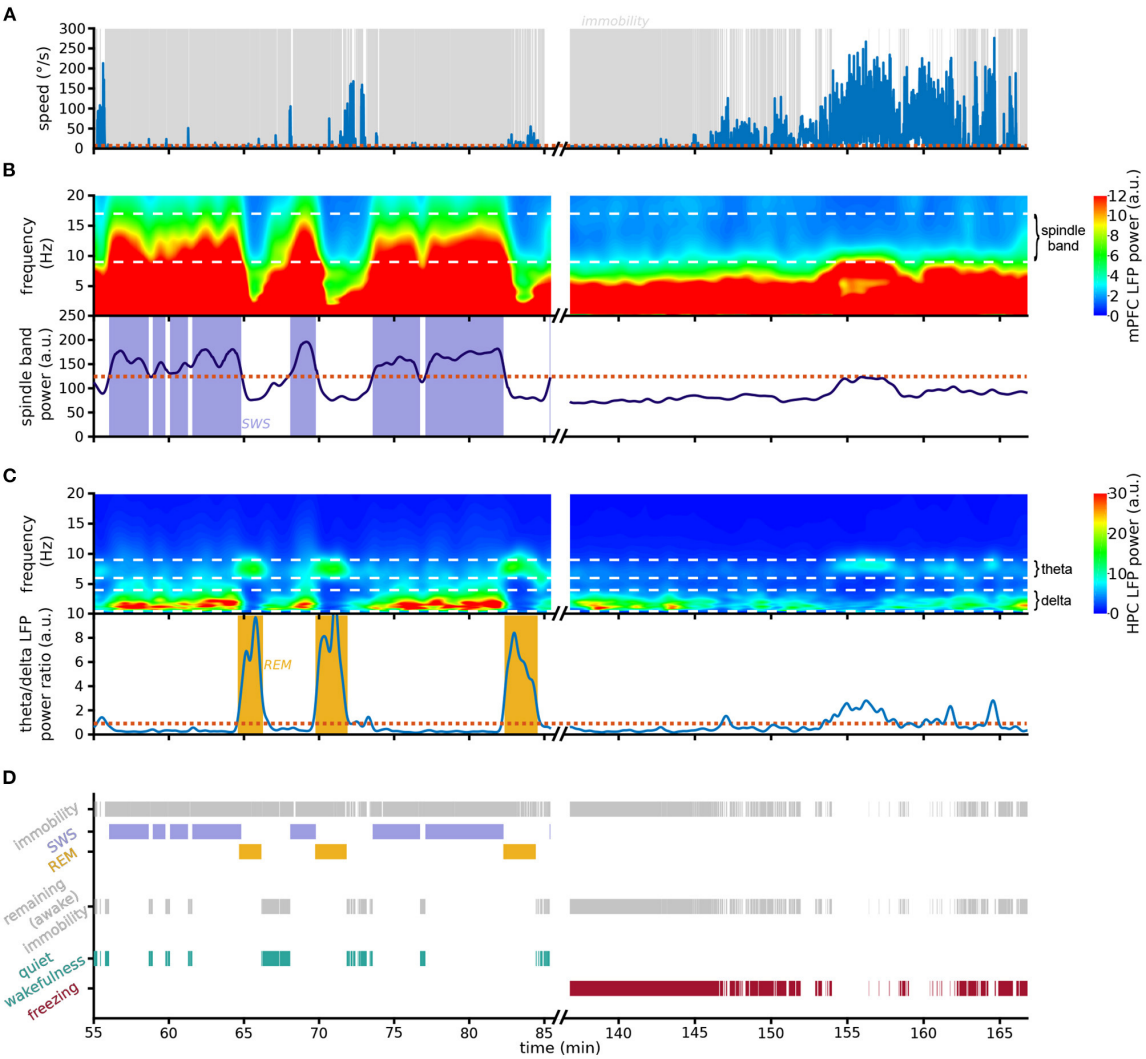
Scoring pipeline. Left: An example 30 min recording in the resting environment; right: an example 30 min recording in context A during the first day of extinction. **(A)** Animal speed as measured by head movements. **(B)** (Top) Spectrogram of mPFC LFP activity; white dashed horizontal lines: boundaries of spindle frequency band. Note the prominent spindle band activity recorded in the rest epoch but absent in the conditioning environment. (Bottom) Smoothed spindle band power. Red dotted line: threshold used to detect SWS; blue shading: detected SWS periods. **(C)** (Top) Spectrogram of HPC LFP activity; white dashed horizontal lines: boundaries of delta and theta frequency bands. Note that in the resting session theta-dominating periods immediately follow the high-spindle power SWS periods depicted above. (Bottom) Theta/delta ratio; red dotted line: threshold used to detect REM sleep; yellow shading: detected REM sleep periods. **(D)** Detection chart. (Top) All detected immobility, SWS and REM periods. (Middle) Immobility periods not identified as either SWS or REM sleep are labeled awake immobility behaviors. (Bottom) Awake immobility periods immediately preceding SWS are classified as quiet wakefulness (green), and the remaining immobility is scored as freezing (red). Note that freezing was the dominating behavior detected during the first 10 min of exposure to the conditioning context A.

Our recordings included the local field potential (LFP) from the hippocampus (HPC) and medial prefrontal cortex (mPFC). As expected, theta and delta oscillations displayed a marked difference between REM and SWS epochs (Figure 2C). However, the theta/delta ratio did not help separate SWS from freezing, because in both of these epochs delta power dominated over theta power (Figure 1B). This is likely due to the overlap between the delta frequency band and the frequency band of the respiratory-driven cortical oscillation around 4 Hz associated with freezing (Ciatipis et al., 2014; Karalis et al., 2016; Lockmann et al., 2016; Biskamp et al., 2017; Moberly et al., 2018; Bagur et al., 2021; Karalis and Sirota, 2022). On the other hand, power in the spindle band (9–17 Hz) was high in sleep epochs and low in freezing (Figure 1B), suggesting that spindle power may be an exclusive marker for SWS.

### 2.2. Smoothed Cortical Spindle Power to Detect SWS

To develop a method to separate freezing and sleep, we took advantage of the lack of overlap between the spindle frequency and the slower 4 Hz oscillation associated with freezing. On the fine timescale, spindle power increases particularly during individual spindles, which are distinct thalamocortical events occurring in SWS. However, spindle events are common in SWS, and therefore the *smoothed* spindle power is relatively constant as it joins multiple spindle events (Supplementary Figure 1). Indeed, the smoothed spindle power during immobility periods follows a bimodal distribution (Supplementary Figure 2), allowing the separation between SWS and non-SWS immobility periods as they are characterized by high and low smoothed spindle power, respectively.

The pipeline we developed (see Section 4 for details) starts by detecting all periods of immobility (Figure 2A). We then classify immobility periods with high spindle power as SWS (Figure 2B). Since REM periods do not occur in isolation but are nested within SWS cycles (McCarley, 2007), we label as REM sleep those epochs where HPC theta power is higher than HPC delta power, on the condition that they closely follow SWS (Figure 2C). In the absence of HPC recordings the algorithm can detect REM using the theta/delta ratio of the cortical LFP used to detect SWS (Supplementary Figure 3).

The remaining immobility periods are considered non-sleep immobility states (Figure 2D). These include the periods of quiet wakefulness prior to falling asleep, which must be disentangled from freezing. We therefore classify these SWS-preceding immobility periods as quiet wakefulness epochs, and the remaining immobility is labeled as freezing (Figure 2D). Contrary to a compounded use of the standard approaches for sleep and freezing detection, this method is designed to guarantee zero overlap between different behavioral states, in particular between freezing and sleep.

### 2.3. Effective Detection of Sleep and Freezing

**Figure 3:**
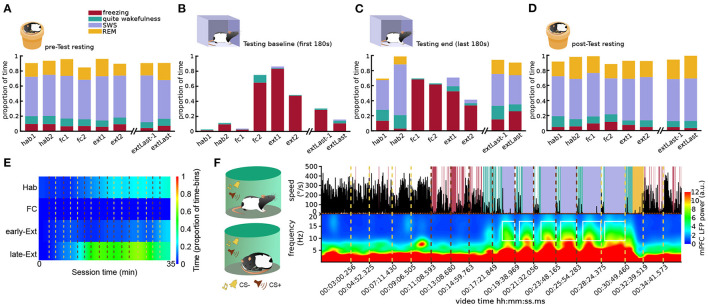
Scoring pipeline performance on a multi-day fear conditioning and extinction protocol. **(A)** Average time classified SWS, REM, quiet wakefulness, and freezing during the rest sessions before exposure to the testing environments for each day of the behavioral protocol. **(B)** Same as **(A)** but for the first 3 min in the conditioning context A. **(C)** Same as **(A)** but for the last 3 min in the conditioning context A. **(D)** Same as **(A)** but for rest sessions following exposure to the testing environments. **(E)** Average time spent sleeping across auditory cued extinction training sessions in context B for all animals; red dashed vertical lines: CS+ onsets; ochre dashed vertical lines: CS− onsets. **(F)** State scoring in a representative session of late extinction of cued fear involving both sleep and freezing (see Supplementary Video 5). (Top) Animal speed as measured by head movements; red dashed vertical lines: CS+ onsets; ochre dashed vertical lines: CS− onsets. Time stamps of CSs presentations corresponding to those overlaid in Supplementary Video 5 frames are indicated below the plots. Shaded area: periods classified as freezing (red), quiet wakefulness (green), SWS (blue), and REM (yellow). Note how the animal did not freeze during CS− presentations, but the onsets of the first three CS+ triggered freezing responses. (Bottom) Spectrogram of mPFC LFP activity; white squares: intervals of elevated sleep spindle power scored as SWS. The rat started to sleep after the fourth CS+ presentation and from then on, every CS+ presentation woke him up, but he fell asleep again within 30–40 s.

To validate our scoring pipeline, we analyzed data from a multi-day fear conditioning and extinction dataset. The results of the algorithm were consistent with behavior to be expected in our protocol. Regardless of the day, in rest sessions animals spent most of the time sleeping, and freezing levels were low (Figures 3A,D). In habituation sessions, in the first 3 min (baseline) of being placed in context A, animals tended to actively explore the environment (Supplementary Video 3), and freezing and sleep were mostly absent (Figure 3B). Conversely, in the last 3 min of these sessions, after the animal had remained there for some time (each training session lasted 35 min), sleep was not uncommon (Figure 3C; Supplementary Video 4). Following fear conditioning in context A, animals tended to freeze there during baseline (Supplementary Video 2). Unlike habituation training sessions, they did not sleep at the end of conditioning ones (Figure 3C). Both of these tendencies (to freeze during baseline and to remain awake until the end of the session) gradually decreased over extinction (Figures 3B,C). Indeed, an animal's tendency to sleep in training sessions appeared to be a good indicator of global fear levels in our task (Figures 3C,E; Supplementary Figure 4). The epochs detected by our approach are therefore consistent with: (1) contextual freezing emerging only after conditioning and decreasing over extinction training and (2) animals sleeping (and therefore being calm) in the conditioning context only at low fear stages of the protocol (before conditioning and in late extinction).

The need for a method separating between sleep and freezing is most pronounced in cases when it's possible to observe both of these behaviors in the same sessions, which can sometimes involve abrupt transitions between states. To test the performance of our method in such a scenario, we analyzed the moments when an animal slept when a CS+ was being presented in cued fear extinction sessions. Indeed, the unanticipated presentations of a strong sound can wake up the sleeping animal. In the example in Figure 3F; Supplementary Video 5, the first couple of CS+ presentations induced freezing behavior, which was detected by our method because smoothed spindle power remained low during these immobility periods. After some minutes, the animal fell asleep, which was accompanied by increased smoothed spindle power. Each subsequent CS presentation briefly woke the animal up, resulting in sleep-wake-sleep transitions detected by our method. The smoothed spindle power can therefore serve as a precise tag for SWS.

Finally, while ground truth data for the internal state of the animal is impossible to obtain, we compared the results of the algorithm to the scoring by an expert observer, blind to the algorithm results, who manually labeled 2 s video snippets (*n* = 600) classified as either freezing or sleep by the algorithm across five recording sessions from three different animals. The freezing vs. sleep separation by this manual scoring agreed with the algorithm classification 92% of the time.

## 3. Discussion

Discriminating between freezing and resting states can be critical in longitudinal recordings of emotional behavior. However, traditionally, these behaviors have been studied separately, and standard scoring methods using immobility and theta/delta ratio cannot disambiguate between them. Unlike delta band power, the smoothed spindle power in cortical LFP recordings can correctly identify SWS epochs and separate them from freezing. The remaining immobility periods can be scored as REM sleep, quiet wakefulness, or freezing, depending on the theta/delta ratio and their timing to detected SWS epochs. We applied this approach to recordings of rats involving both freezing and sleep as part of a fear conditioning and extinction protocol, and our results are consistent with correct scoring of freezing and sleep states.

The key advantage of the smoothed spindle power is that while it is high in SWS, it remains low during freezing. Basing the SWS detection on this signal alone thus avoids confounding SWS and freezing, when other signals such as delta power can respond strongly to both behaviors due to the overlap between the delta band and the 4 Hz oscillation (2–5 Hz respiratory-driven rhythmic activity; Moberly et al., 2018). While the spindle band power is used by many sleep classification algorithms (e.g., Gottesmann et al., 1977; Louis et al., 2004; Liang et al., 2012; Supratak et al., 2017), its contribution is often secondary to slow oscillations in the delta band. Therefore, these algorithms can be used to score sleep stages only after freezing has already been excluded, but they were not designed to separate between sleep and freezing, especially given their assumption that all immobility corresponds to resting states.

Recently, Bagur et al. (2018) showed that sleeping and freezing states can be disambiguated using oscillations recorded in the olfactory bulb. This is a powerful method which resolves the sleeping/freezing ambiguity. Using the neocortical smoothed spindle power as proposed here can be an alternative in the absence of such recordings from the olfactory bulb.

Here, we introduced a technique to detect SWS periods based solely upon the smoothed spindle band oscillatory activity and we demonstrated its effectiveness in our data. To our knowledge, this is the only automated approach discriminating between freezing and resting immobility in the absence of olfactory bulb recordings. Our algorithm is straightforward to implement as it only requires recording from a single neocortical electrode, and it could be of use for future systems neuroscience research involving both fear and rest behavioral states. Moreover, the lack of overlap between the spindle band and breathing frequencies observed in immobility (Herent et al., 2020) can help separate sleep from other awake behaviors beyond freezing, permitting the smoothed spindle power to serve as a simple method to detect SWS in general.

## 4. Materials And Methods

### 4.1. Recording of Neural Activity During a Multi-Day Fear Conditioning and Extinction Training Protocol and Peri-Training Rest

#### 4.1.1. Animals

Five male Long-Evans rats (350–400 g at the time of the surgery, 2–5 months old) were housed individually in monitored conditions (21°C and 45% humidity) and maintained on a 12 h light–12 h dark cycle. In order to avoid obesity, feeding was restricted to 13–16 g chow per day, with water available *ad libitum*. To habituate the rats to human manipulation, they were handled each workday. All experiments conformed to the approved protocols and regulations of the local ethics committee and the French ministry of agriculture and the French ministry of higher education and research.

#### 4.1.2. Surgery

Rats were deeply anesthetized using a ketamine-xylazine mixture (Imalgene and Rompun, 180 and 10 mg/kg, respectively) and anesthesia was maintained with isoflurane (0.1–1.5% in oxygen). Analgesia was assured by subcutaneous injection of buprenorphine (Buprecaire, 0.025 mg/kg) and meloxicam (Metacam, 3 mg/kg). The animals were implanted bilaterally with a custom built microdrive with 24–42 bundles of independently movable twisted electrodes (12 μm tungsten wires twisted in groups of either six or eight wires and gold-plated to 200 kΩ) 0.5 mm above the target brain regions. Electrode bundle placement varied between rats. Nevertheless, in all animals there were electrodes implanted in the medial prefrontal cortex (sterotaxic coordinates: ±0.3–0.6 mm mediolateral and +2.5–4.8 mm anterioposterior from bregma) and dorsal hippocampus (±3.8–5 mm mediolateral and −5 mm anterioposterior from bregma). During recovery after surgery (minimum 7 days), the rats received antibiotic (Marbofloxacine, 2 mg/kg) and analgesic (Meloxicam, 3 mg/kg) treatments via subcutaneous injections, and were provided with food and water *ad libitum*. The recording electrodes were then progressively lowered until they reached their targets.

#### 4.1.3. Behavioral Apparatus

After full recovery, rats were exposed to two testing environments (context A and context B), and one resting environment. Context A was a cubicle conditioning chamber (40×40×40 cm) with gray plexiglass walls lined with ribbed black rubber sheets and a floor composed of nineteen stainless steel rods [0.48 cm diameter with 1.6 cm spacing connected to a scrambled shock generator (ENV-414S, Med Associates, USA)]. It was mildly scented daily with mint-perfumed cleaning solution (Simple Green, Sunshine Makers). Context B was a stadium-shaped PVC enclosure (30 cm side and 15 cm radius) with a black wooden floor and walls lined with light brown pieces of rope rug. It was mildly scented daily with a vanilla extract solution. The resting environment was a cloth-lined plastic flowerpot (30 cm upper diameter, 20 cm lower diameter, 25 cm high). Auditory stimuli (conditioned stimuli, CSs) were delivered in either of the two testing environments via a custom-made electronic system. The stimuli were white noise (CS+) or 8 kHz pure tone (CS−) 250 ms long pips repeated at 1 Hz for the duration of 20 s at 80 dB.

#### 4.1.4. Behavioral Protocol

Habituation took place on days 1 and 2. On day 1, CSs were presented in one context and on day 2 they were presented in the other (context order presentation was switched every day and was counterbalanced across animals), habituating the animals to the stimuli. On days 3 and 4, the animals were fear conditioned in context A, where CS+ presentations were coupled with foot shocks (1 s, 0.6 mA, co-terminating with CS+ presentations). Extinction training began on day 5, with CS presentations in context B and context exposure (no CS presentations) in context A. Extinction training was repeated every day until the rat was seen sleeping throughout CS+ presentations.

Every day of the experimental protocol consisted of one 35 min session in each context. When introduced in the contexts the animals were either presented with the auditory CSs (after a baseline period of 3 min, the animals were presented to 16 CSs, 8 CS+, and 8 CS−, separated by inter-trial intervals of random duration, range 120–240 s) or received no auditory stimuli (context exposure sessions). During habituation and fear conditioning, CS+ and CS− were presented in pseudorandom order (no more than 2 consecutive presentations of the same-type CS), while in extinction training, 4 CS− were presented first, followed by 8 CS+ and then 4 CS−. Before and after each session the animals were left undisturbed for at least 2 h in the resting environment to record sleep activity.

#### 4.1.5. Data Acquisition and Processing

An inertial measurement unit (IMU, non wireless version of the one described in Pasquet et al., 2016) recorded 3D angular velocity and linear acceleration of the animals' heads (sampled at 300 Hz). Animal behavior was also recorded by lateral video cameras (50 Hz sampling rate) in contexts A and B (acA25000, Basler). Brain activity was recorded wideband at 20,000 Hz using a KJE-1001 data acquisition system (Amplipex, Szeged, Hungary). Neurophysiological and behavioral data were explored with NeuroScope (Hazan et al., 2006). LFPs were derived from wideband signals by downsampling all channels to 1,250 Hz. At the end of the experiments, recording sites were marked with small electrolytic lesions (~20 μA for 20 s, one lesion per bundle). After a delay of at least 3 days to permit glial scarring, rats were deeply anesthetized with a lethal dose of pentobarbital and intracardially perfused with saline (0.9%) followed by paraformaldehyde (4%). Coronal slices (35 μm) were stained with cresyl-violet and imaged with conventional transmission light microscopy (Supplementary Figure 5). Recording sites were reconstructed by comparing the images with the stereotaxic atlas of Paxinos and Watson (2013).

### 4.2. Data Analysis and Statistics

Data were analyzed using FMAToolbox (http://fmatoolbox.sourceforge.net), Chronux (http://chronux.org/), and custom written programs in Matlab (MathWorks, Natick, MA).

#### 4.2.1. Scoring of Behavioral States With Standard Approaches

In order to compare our proposed approach with standard sleep and freezing detection protocols, we performed standard behavioral scoring as follows. Automatic detection of immobility was performed by applying a threshold detection routine to the angular speed calculated from gyroscopic data as we described previously (Pasquet et al., 2016).

##### 4.2.1.1. Freezing Detection

Typically, the minimum duration of immobility to be classified as freezing ranges between 0.5 and 2 s, and brief movements of 0.1–0.2 s are ignored (e.g., Courtin et al., 2014; Jercog et al., 2021). We therefore defined freezing as moments where the animals remained immobile for more than 1.5 s, ignoring brief movements of <0.2 s.

##### 4.2.1.2. Sleep Detection

To identify sleep stages (NREM and REM), LFP data was visualized using Neuroscope (Hazan et al., 2006) and a hippocampal channel with clear theta oscillations during exploration was identified. The signal from this channel was filtered in the theta (6–9 Hz) and delta (0.5–4 Hz) bands. Typically, only periods longer than 30–120 s are retained as sleep and periods around brief movements of 0.5–1 s are merged together (e.g., Drieu et al., 2018; Todorova and Zugaro, 2019). Therefore, here, only the periods of immobility lasting longer than 60 s were considered, and brief movements shorter than 1 s were ignored. The time bins of theta/delta ratio of these periods were clustered in two groups with k-means.

#### 4.2.2. Scoring of Behavioral States With Spindle Power

##### 4.2.2.1. Slow Wave Sleep Detection

Immobility was detected as above. LFP data was visualized using Neuroscope (Hazan et al., 2006) and a channel with distinct spindles during sleep (mPFC) was selected. The LFP signal was filtered in the spindle band (9–17 Hz), and the instantaneous amplitude was estimated using the Hilbert transform. This amplitude was then smoothed (Gaussian window of 14 s), and immobility periods where the smoothed amplitude exceeded a k-means identified threshold (Supplementary Figure 2) were classified as SWS. The minimum duration allowed for sleep bouts and the maximum duration of small movements are parameters that users are free to select. For the results presented here these parameters were set to 30 and 1 s, respectively.

##### 4.2.2.2. REM Sleep Detection

The remaining immobility periods where the ratio between the theta (6–9 Hz) and delta (0.5–4 Hz) power of the HPC LFP signal exceeded 1 and followed a SWS period were classified as REM. The maximum delay by which REM periods can follow SWS is a parameter set by the user. However, since SWS to REM transitions are known to last up to 30 s (Datta and Hobson, 2000), we recommend allowing for at least 30 s.

In the absence of hippocampal LFP the algorithm can detect REM periods using the theta/delta ratio of the cortical LFP used to detect SWS, with the following adjustments. First, the cortical theta/delta ratio is smoothed by Gaussian smoothing window, with a window width of 8 s maximizing the correspondence to the results obtained using HPC recordings. Then, because the theta/delta ratio tends to be lower in the mPFC than in the hippocampus, the threshold to detect REM epochs is determined using the Otsu method (Otsu, 1979; which divides the theta/delta values into two groups maximizing inter-class variance) rather than the hard-coded threshold of 1 used for HPC recordings. The resulting REM periods thus obtained by PFC recordings matched the ones estimated by HPC recordings 93.45% of the time (Supplementary Figure 3).

##### 4.2.2.3. Quiet Wakefulness Detection

Animals don't fall asleep in their freezing posture: at the minimum, they would always readjust their heads and more typically also curl up their bodies. This means that freezing and pre-sleep immobility are interrupted by movement. Therefore, other periods of immobility which preceded SWS by <2 min were classified as immobile wakefulness. This duration is a parameter free to user selection, and reasonable choices would fall within a range of 30 s to a few minutes.

##### 4.2.2.4. Freezing Detection

All the remaining immobility was classified as freezing with a minimum duration of freezing bouts and minimal interruptions set respectively to 2 and 0.2 s. These parameters are free to user selection in our code.

#### 4.2.3. Analyses of Scoring Performance and Data Visualization

To assess the optimal smoothing window of the spindle power, we computed the effectiveness metric *m* of the k-means separation of the smoothed spindle power in two groups, as defined by the proportion of inter-group variance and computed as $m=1-\frac{\sigma_{i}}{\sigma_{tot}}$, where σ_*i*_ is the intra-group variance σ_*tot*_ is the total variance of the smoothed spindle power.

To compute spectrograms, we employed an adapted version of the wavelet transform code from http://paos.colorado.edu/research/wavelets/.

## Code Availability Statement

A Matlab function to apply the scoring pipeline presented here is freely available on github (https://github.com/mnpompili/behavioralStates.git).

## Data Availability Statement

The data supporting the conclusions of this article will be made available by the authors upon request.

## Ethics Statement

The animal protocol was reviewed and approved by Comité d'Ethique en Matière d'Expérimentation Animale N°59.

## Author Contributions

MP and RT: research project conception and design and data analysis. MP: funding gathering and project management. RT: algorithm conception and implementation. MP with contributions from RT: manuscript. Both authors contributed to the article and approved the submitted version.

## Funding

MP was supported by the Agence Nationale de la Recherche (ANR-20-NEUC-0005-01) and RT by Cornell University.

## Conflict of Interest

The authors declare that the research was conducted in the absence of any commercial or financial relationships that could be construed as a potential conflict of interest. The handling editor declared past co-authorships with both authors.

## Publisher's Note

All claims expressed in this article are solely those of the authors and do not necessarily represent those of their affiliated organizations, or those of the publisher, the editors and the reviewers. Any product that may be evaluated in this article, or claim that may be made by its manufacturer, is not guaranteed or endorsed by the publisher.

## Supplementary Material

Supplementary Video 1 Representative video of a rat sleeping in the resting environment. The video corresponds to the time interval shown on the left side of Figure 1B, part of the resting session immediately preceding the first contextual extinction session. Note how the animal sleeps almost all the time with small movements to adjust its posture.

Supplementary Video 2 Representative video of a rat freezing in the conditioning environment. The video corresponds to the time interval shown on the right side of Figure 1B, that is the baseline period of the first contextual extinction session. These are the first moments the animal enters this environment since conditioning. Note how the animal freezes all the time in a stiff position and only the movements associated to a deep respiration are visible.

Supplementary Video 3 Representative video of an animal exploring the conditioning environment. This corresponds to the first 30 seconds of exposure to the conditioning environment on the fourth day of extinction. This is the same rat of Supplementary Video 2 but after 3 sessions of contextual fear extinction. Note how the animal does not freeze any more and explores the environment as the contextual fear has been extinguished.

Supplementary Video 4 Representative video of an animal sleeping in the conditioning environment. This corresponds to 30 seconds in the conditioning environment on the fourth day of extinction. Namely, the same session of Supplementary Video 3 15 minutes later. Note how the animal sleeps, although uncomfortably, on the rods of the floor. The reclined head adjusted on the fore-paws is a good sleep marker.

Supplementary Video 5 Video corresponding to the example session of Figure 3F where both freezing and sleeping were detected. The timestamp overlaid on video frames are on the same time scale of the x axis of the figure. Note how the animal explores the environment during baseline, freezes after the onsets of the first two CS+ presentations, but then gradually falls asleep. This rest is systematically interrupted by CS+ presentations (only two episodes shown in this video). All these transitions were captured by our technique as shown in Figure 3F.
