# Supplementary material for: Discriminating Sleep From Freezing With Cortical Spindle Oscillations
Source: Front Neural Circuits. 2022 Mar 24;16:783768. doi: 10.3389/fncir.2022.783768 (PMC8988299; doi:10.3389/fncir.2022.783768)
Supplement: Supplementary file 2 [file Data_Sheet_2.pdf]

SUPPLEMENTARY FIGURES

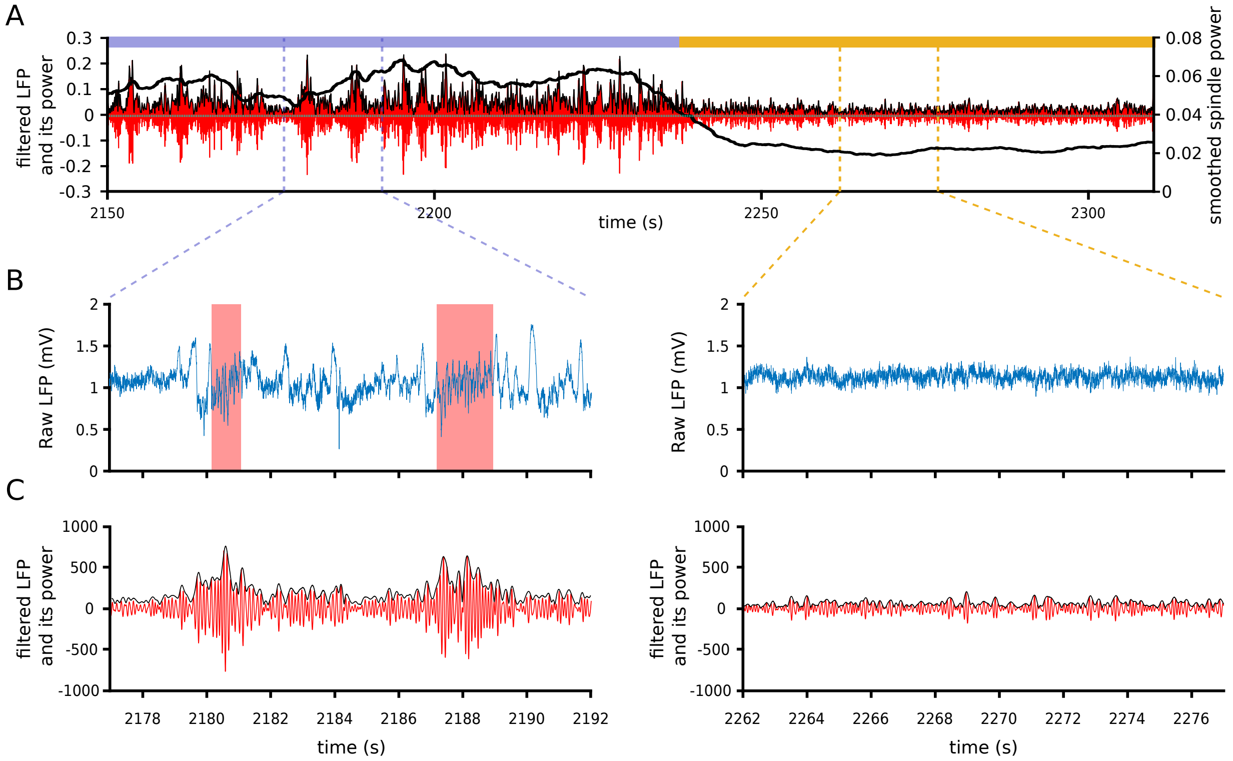

Supplementary Figure 1. Smoothed spindle power links distinct spindle events over time. (**A**) mPFC LFP filtered in the spindle frequency band (red) and its power (black) during a representative SWS-to-REM transition. The thicker black curve is the smoothed spindle power (right y axis) while the dashed green line depicts the SWS detection threshold. Blue and yellow bands above mark the periods identified as SWS and REM, respectively. Vertical dashed lines: the boundaries of the example intervals in B and C. (**B**) Representative examples of raw PFC LFP during SWS (left) and REM (right) during the intervals marked in A. The two representative spindles are marked by the red rectangles. (**C**) Representative examples of the PFC LFP filtered in the spindle frequency band (red) and its power (black).

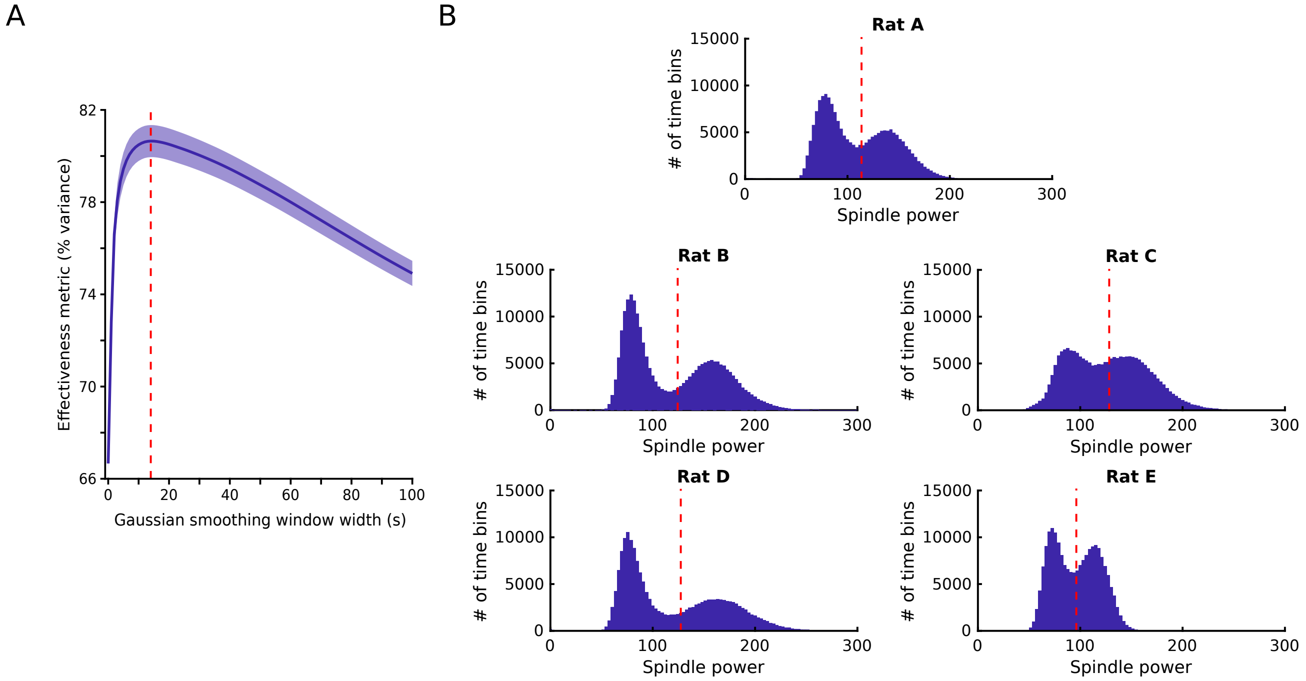

Supplementary Figure 2. Separation of periods with high vs. low spindle power. (**A**) Effectiveness of separation of high vs. low spindle power periods as a function of smoothing window width. The curve peaks at 14 seconds (red dashed line). (**B**) Bimodality of smoothed (window of 14 s) spindle power distributions in all subjects. Red dashed vertical lines indicate individual spindle power thresholds obtained with k-means.

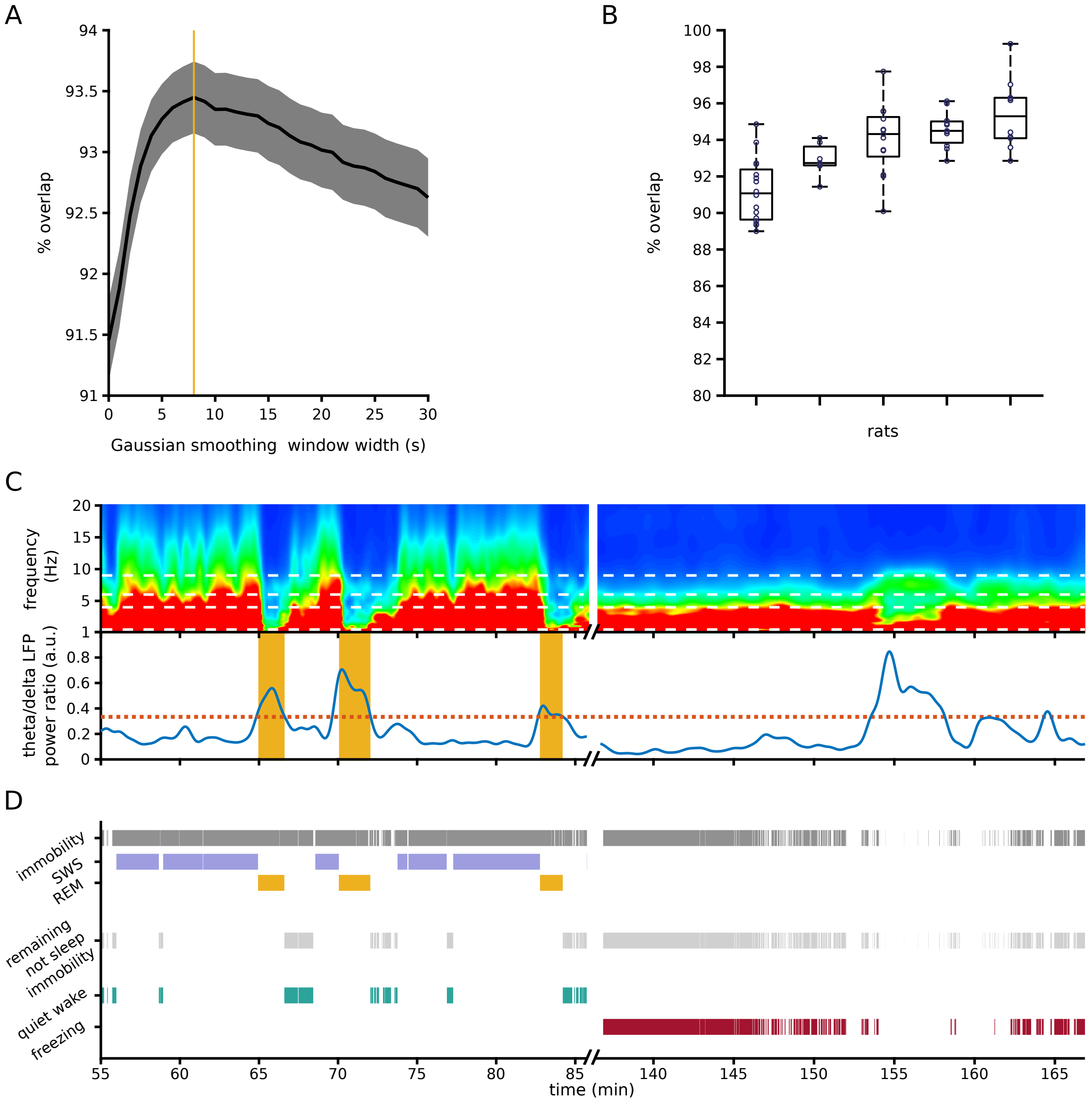

Supplementary Figure 3. Detection of REM sleep with theta/delta ratio signal from the PFC. (**A**) Overlap between REM periods detected using HPC LFP and REM periods detected using PFC LFP of the same cortical channel used for SWS detection as a function of smoothing of the PFC theta/delta ratio. Solid line: average across 57 recording sessions. Shaded area: SEM. Note that the optimal overlap was obtained using a Gaussian window of 8 s (vertical yellow line). (**B**) Overlap between HPC and PFC-obtained REM periods as in (A) across all recorded sessions, with the PFC theta/delta ratio smoothed with Gaussian window at 8 s. Circles: individual sessions; box and whisker plots: median and 25th and 75th percentiles. (**C,D**) Example of REM detection with PFC smoothed theta/delta. Same example as in Fig. 2.

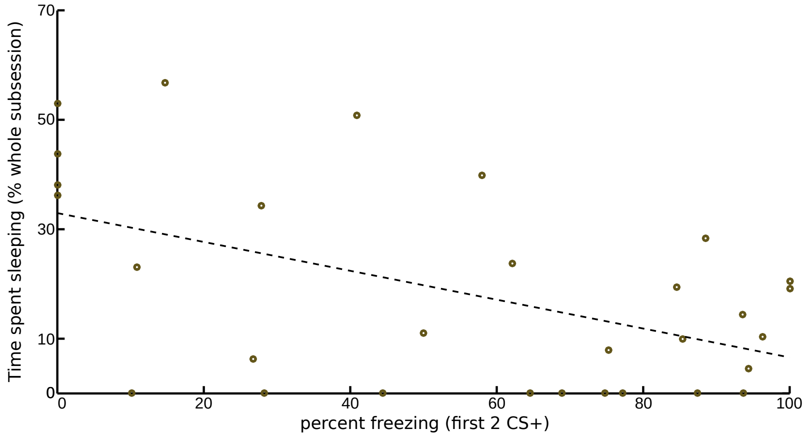

Supplementary Figure 4. Sleeping during training is an indicator of calmness. Correlation between the time spent freezing during the first two CS+ presentations and time spent sleeping in cued extinction sessions. Sleep time and freezing time are significantly anti-correlated (Spearman rank test, $p<0.001$).

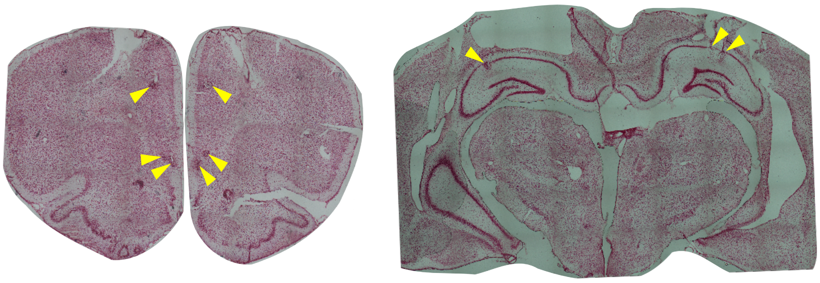

Supplementary Figure 5. Representative recording positions in the mPFC (left) and dorsal HPC (right).
